# A Multispectral Camera Development: From the Prototype Assembly until Its Use in a UAV System

**DOI:** 10.3390/s20216129

**Published:** 2020-10-28

**Authors:** Alejandro Morales, Raul Guerra, Pablo Horstrand, Maria Diaz, Adan Jimenez, Jose Melian, Sebastian Lopez, Jose F. Lopez

**Affiliations:** Institute of Applied Microelectronics (IUMA), University of Las Palmas de Gran Canaria, 35003 Las Palmas de Gran Canaria, Spain; amorales@iuma.ulpgc.es (A.M.); rguerra@iuma.ulpgc.es (R.G.); phorstrand@iuma.ulpgc.es (P.H.); mdmartin@iuma.ulpgc.es (M.D.); ajimenez@iuma.ulpgc.es (A.J.); jmelian@iuma.ulpgc.es (J.M.); lopez@iuma.ulpgc.es (J.F.L.)

**Keywords:** multispectral camera, CMOS sensor, UAV

## Abstract

Multispectral imaging (MI) techniques are being used very often to identify different properties of nature in several domains, going from precision agriculture to environmental studies, not to mention quality inspection of pharmaceutical production, art restoration, biochemistry, forensic sciences or geology, just to name some. Different implementations are commercially available from the industry and yet there is quite an interest from the scientific community to spread its use to the majority of society by means of cost effectiveness and ease of use for solutions. These devices make the most sense when combined with unmanned aerial vehicles (UAVs), going a step further and alleviating repetitive routines which could be strenuous if traditional methods were adopted. In this work, a low cost and modular solution for a multispectral camera is presented, based on the use of a single panchromatic complementary metal oxide semiconductor (CMOS) sensor combined with a rotating wheel of interchangeable band pass optic filters. The system is compatible with open source hardware permitting one to capture, process, store and/or transmit data if needed. In addition, a calibration and characterization methodology has been developed for the camera, allowing not only for quantifying its performance, but also able to characterize other CMOS sensors in the market in order to select the one that best suits the budget and application. The process was experimentally validated by mounting the camera in a Dji Matrice 600 UAV to uncover vegetation indices in a reduced area of palm trees plantation. Results are presented for the normalized difference vegetation index (NDVI) showing a generated colored map with the captured information.

## 1. Introduction

In the last few decades, multi and hyperspectral imaging have made enormous progress due to the improvements achieved in electronics, computation and software, becoming a very powerful tool for acquiring information relevant to many different fields [1,2,3,4]. It is in the remote sensing area where it has found most of its use [5,6], both with sensors mounted onboard satellites [7,8,9] as well as onboard unmanned aerial vehicles (UAVs) [10,11,12]. The latter has been gaining more and more attention as it presents the following attractive advantages: A flexible revisit time, a better spatial resolution, which permits a deeper and more accurate data analysis, and a lower overall solution cost, which has permitted small research groups to start developing their own platforms [13].

Nonetheless, the incorporation of multi and hyperspectral cameras into drones has only been possible after a great deal of efforts has been invested in the miniaturization of these devices. The emergence of new alternatives in the technology for constructing the three dimensional (3D) image cube out of the standard two dimensional (2D) sensor array has certainly paved the way forward [14,15]. Back in the times where this sensing technology was exclusively installed in satellites or airplanes, whiskbroom [16] and pushbroom imagers [17] were the most recurrent devices to be used, mainly relying on a complex optics system to construct the hypercube.

Nowadays, there is a wide range of choices when it comes to selecting the camera to utilise, besides the already mentioned technologies. To name some: multi-camera 2D imagers [18,19], with as many 2D sensors as wavelengths are captured; sequential band systems [20], recording bands or sets of bands sequentially in time; multi-point spectrometers [21], using a beam splitter to divide the spatial image into sections of which the signal is spread in the spectral domain; mosaic filter-on-chip [22], where each sensor pixel carries a spectral filter built on chip and spatio-spectral cameras, consisting of a continuous filter in front of the 2D panchromatic sensor. Each of the aforementioned solutions present some advantages and disadvantages over each other, the specific application at hand being the one that tips the balance in favour of one or another.

As a matter of fact, some of the mentioned technologies are more suitable for acquiring hyperspectral information as they are able to capture hundreds of bands per pixel, while others can just reach up to a dozen of bands, forming what is called a multispectral image. However, it is not always necessary to acquire a vast amount of spectral information per pixel, since there are certain applications where the acquisition of just a few bands is enough. For instance, in the scientific literature different applications can be found benefiting from the multispectral images captured onboard UAVs. In [23,24], fire outbreaks are detected in forests and burn severity evaluated. A water quality analysis of small reservoirs is presented in [25]. In [26], the algae bloom in shallow water problem is constantly monitored and the evolution evaluated. However, it is in agriculture where multispectral sensors relying on UAVs has been strongly positioned as a field of application [27], contributing to what has been termed *smart farming*. The use of UAVs equipped with the appropriate sensors permits a periodical monitoring of plants during their cultivation, being a step ahead in applying a special treatment to the plant may need before it is too late. In this scenario, several multispectral sensors are being widely used on-board UAVs for collecting spectral information that allows for the generation of maps of a certain area for indicating the plant state and evolution [28]. One of the most typical examples is the normalized difference vegetation index (NDVI), which indicates the vigour of the plants using the information of two spectral channels placed in the red and near infrared parts of the electromagnetic spectrum [29].

Nevertheless, there are many more indices, in addition to the NDVI index, that provide useful information for smart farming applications [30]. These indices use the spectral information corresponding to different parts of the electromagnetic spectrum. Due to this, it is certainly desirable for the camera to be mounted onboard the UAV platform to have a certain degree of flexibility in the spectral bands to be captured in order to be adapted to different needs within a specific field and even provide services to other domains.

In this work, a multispectral camera prototype is presented, capturing images in a sequential fashion with a short time delay between the acquisition of each individual spectral band. The camera is based on an single image sensor and a rotating wheel holding the optic filters that produce each of the captured wavelengths. In order to set the wheel in motion, a system based on a stepper motor controlled by an Arduino board has been devised. A single board computer is in charge of coordinating both the sensor and the wheel, in order to acquire proper images.

The use of the sequential capturing mode has been used as it provides the flexibility of selecting the desired number of bands by just replacing the wheel, or even add an extra one. Moreover, the whole design requires just one image sensor which is usually the cost driver in such devices, thus, presenting a low cost solution.

Once the sequential approach has been selected, another key aspect to take into account in the design is the position of the filters in the optical path. In this regard, there are mainly two options where they can be placed: in between the lens and the sensor or right in front of the lens. While some authors in the literature have adopted the first option [31,32,33], as this allows a more robust design and the possibility of using smaller and cheaper filters, in this work we have opted for the second solution, which brings up some advantages to the whole system. In fact, modifying the optical path of the lens-sensor system carries more aberrations than placing the filters before the lens. This has been thoroughly discussed in [34], where the aberrations have been mathematically modelled to compensate for in a post-processing phase. Furthermore, having the wheel as the outer element provides higher accessibility to individual filter elements, enabling their easy replacement, and furnishes the system with flexibility as extra layers can be added—for instance, another filter wheel to increase the number of bands and the spectral capabilities of the camera.

In addition, the camera has been developed as a set of layers stacked on top of each other, going a step further in the modularity concept. This way, the component replacement is straight forward should, for instance, another single board computer with higher computational capabilities be required, or another sensor based on its cost or added features.

Having a single board computer as a coordinator of the tasks can be further exploited to perform onboard processing within the platform and obtain immediate results that are imperative for real time applications, or even apply compression to the images [35,36] in order to transmit them to the ground more efficiently. As the camera design is mainly intended to be mounted onboard a UAV, the single board computer from the camera can be used as well to plan, monitor and supervise the platform flight mission. This substantially reduces the complexity of the whole flying platform, which is yet another advantage of the proposed solution.

Furthermore, in this work, an exhaustive spectral characterisation of the camera was performed, as opposed to other state-of-the-art multispectral implementations where the spectral response of the device is not proven [37,38]. In this sense, it is worth mentioning that others evaluate the spectral response on-site, placing well characterised materials on the ground to capture them during the flight [39]. In our case, the characterisation was performed in a more controlled environment as it is our hyperspectral laboratory, which includes materials whose spectral response is well known, a Headwall Photonics [40] light consisting of a halogen bulb with a relatively homogeneous spectral emitting signal, and a rail pointing downwards where the camera can be fixed, all within a sealed cage.

The rest of the manuscript is organised as follows. Section 2 describes the proposed camera development, first outlining each individual hardware element involved in the system (Section 2.1), and secondly, detailing the software implementations carried out to achieve a smooth system functioning (Section 2.2). Section 3 explains the calibration process that is carried out prior to starting to acquire images with the camera, in order to have the largest possible dynamic range. In Section 4, the camera characterisation procedure in the laboratory using material whose spectral signature is well known in advance is presented. In Section 5, the obtained results are displayed, first comparing the performance of different sensor types in the laboratory, Section 5.1, then some NDVI results of captured images both in the laboratory and onboard a UAV Section 5.2. Finally, Section 6 discloses the obtained conclusions and outlines further research lines.

## 2. Multispectral Camera Development

As mentioned earlier, the approach pursued to accomplish the camera prototype design is mainly based on two key aspects: modularity and low cost. While the first one makes sure each individual component that makes up the whole system can be easily replaced, the second one reduces the cost of the technology, thus making it more appealing for different sectors that could benefit from remote sensing activities.

In order to achieve both goals simultaneously, a design based on a rotating filter wheel was implemented. In this way the number of required sensors is reduced to one. Sensors are usually the cost drivers in a multispectral camera, hence, using a single sensor to capture all wavelengths instead of having one sensor for each filter results in an overall lower cost solution. In addition, the proposed design enables the possibility of easily replacing the optic filters installed in the wheel, change the wheel for another one with a different number of filter placeholders, or even combine more than one wheel to increase the spectral resolution of the device.

On the other hand, the followed approach entails a few disadvantages that should not be disregarded. Having a unique sensor to capture all different wavelengths requires motion to acquire the spectral dimension. For this particular case, the camera frame rate is restricted by the wheel revolutions per minute (rpm), and ultimately it will be reflected in a limitation of the carrying platform speed.

The purpose of this section is to provide all the information details of the carried out design both in terms of hardware components that make up the whole assembly and software programmed to integrate the entire system and correctly operate the camera.

### 2.1. Hardware Development

**Figure 1:**
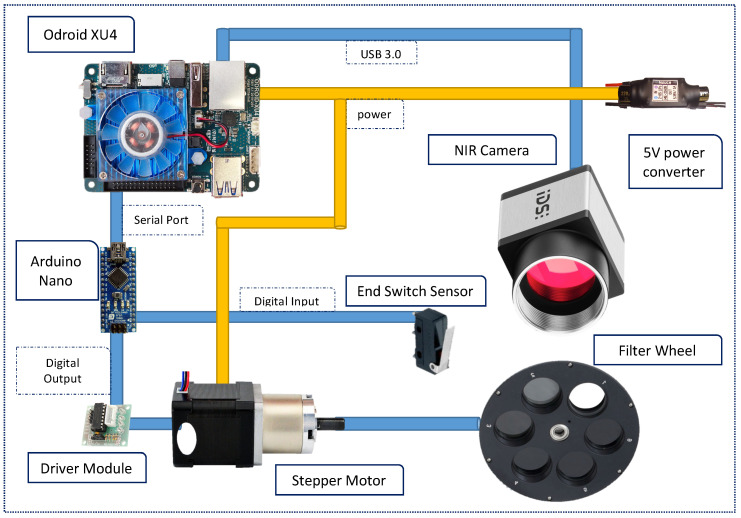
System Diagram.

The system consists of a single CMOS sensor, a filter wheel installed in front of the optics, an electrical system based on a stepper motor that drives the wheel rotation and a power converter that takes care of supplying the right voltage level to every individual element. Finally, a single board computer is used to control and synchronise all processes. The parts have been assembled using a plastic structure that holds the elements together while also provides the necessary coupling structure to install the camera on flying platforms. Figure 1 presents a diagram of the involved hardware elements in the system and their interconnections.

In this section each individual component is explained and the reason for the selection of the particular product argued.

#### 2.1.1. CMOS Sensor

When it comes to select a suitable image sensor sensitive to the radiation in the visible near infrared (VNIR) region of the spectrum, the search is narrowed down to two main technologies: complementary metal oxide semiconductor (CMOS) and charged coupled device (CCD). The advantages and disadvantages of both technologies have been broadly discussed in the literature in the past few decades [41]. Even though CMOS sensors are constantly improving and getting closer to CCD sensors performance, they still have a lower light sensitivity, lower charge capacity and a higher noise, all of this impacting the pixel uniformity [42,43]. On the other hand, CMOS sensors feature a higher frame rate, less power consumption and are considerably cheaper [41,44].

Despite being less expensive than other analogous solutions, the CMOS sensor remains one of cost driver components conforming the multispectral camera. There is a great variety of CMOS sensors in terms of pricing, depending on the manufacturer and its characteristics. Higher resolution or better quality sensors push the price up together with the sensor controllability, as some manufacturers provide a custom application programming interface (API) that gives full accessibility to all sensor parameters and captured raw data.

**Figure 2:**
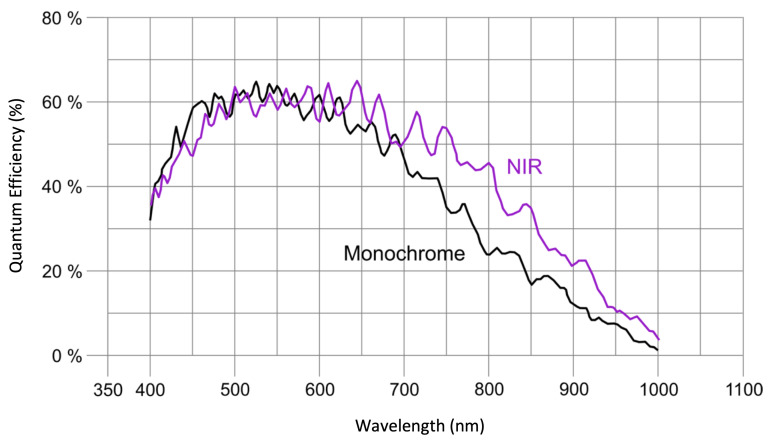
Comparison between the monochrome and the NIR version of the CMV2000-3E12M sensor.

The typical sensitivity of CMOS sensors is approximately between 400–1000 nm [41,45]. Most general purpose cameras today use this type of sensor in order to provide colour images and video. For that purpose, they integrate a so called Bayer filter pattern on top of the sensor in order to obtain the three matrices corresponding to each colour (Red-Green-Blue) out of an individual 2D sensor array. In addition, these cameras add an infrared filter, which prevents the radiation above 750 nm tamper the image. Multispectral and hyperspectral camera devices rely on the CMOS technology as well [46], not only due to the cost factor, but also because the frame rate is a key feature for these devices. In these cameras, the sensor is mounted in its panchromatic version, without Bayer filter and without infrared filter as well, as the radiation above 750 nm is very relevant. Moreover, sensor manufactures usually provide two versions of the panchromatic sensor: monochrome and NIR. They mainly differ in the thickness of the silicon wafer. The NIR type of sensor has a higher thickness, which provides a higher sensitivity in the near infrared section of the electromagnetic spectrum (>800 nm). The sensitivities of both type of sensors are compared in Figure 2.

For all the mentioned reasons, in this work we have opted for a single CMOS near-infrared (NIR) sensor, particularly the CMV2000-3E12M [47] (AMS AG [48], Premstaetten, Austria), integrated in the UI-3360CP-NIR-GL camera (Imaging Development System, IDS [49], Obersulm, Germany) [50]. From here on in the manuscript, we will be referring to this camera device as IDS camera. The CMV2000-3E12M comes with a variation processed on 12 μm epitaxial silicon wafers. The increased thickness in the wafer layer improves the quantum efficiency of the sensor for wavelengths above 600 nm, as indicated by the purple line in Figure 2. The sensor data specification is shown in Table 1. This version of the sensor was selected due to its better performance across the entire spectrum.

#### 2.1.2. Single Board Computer

In order to control the CMOS sensor integrated into the IDS UI-3360CP-NIR-GL camera and trigger it at the desired instance, some sort of processing unit is required in the whole system. This unit would then be in charge not only of controlling the camera, but also positioning the wheel at the right place to acquire images at a specific wavelength.

In order to accomplish the aforementioned tasks, in this work a single board computer is being used for two main reasons. On the one hand, it comes with outstanding processing capabilities that allow for performing tasks onboard, which in turn permits applying any algorithm to the images as they are being captured. This feature is specially attractive for real time applications. On the other hand, as the intention is to use the camera in UAVs, the same board can potentially be used to control the flight, reducing the complexity of the system even more.

There are several options in the market that would fit the proposed design. The Odroid XU4 (Hardkernel Co., Ltd., Anyang, South Corea) has been finally selected as it presents a good trade-off between size, power consumption, computation capabilities and price. Additionally, it comes with all the necessary input and output interfaces, as well as an integrated GPU, which could be useful to speed up some of the on-board processing algorithms. Table 2 shows some basic characteristics of the selected Odroid XU4.

The Odroid XU4 is then in charge of controlling the image capturing process by setting the adequate values to the camera properties, such as exposure time, frame rate and brightness. It does also trigger the capture and stores the acquired images in its memory. Finally, it is in charge of setting the position of the wheel holding the filters. To do so, the Odroid XU4 is connected to an Arduino Nano board via USB and implements a very simple set of commands that are transmitted through the serial protocol. The Nano board is, in turn implemented into the functions to control the stepper motor that drives the wheel. This configuration disengages the single board computer from the wheel motion, which is aligned with the modularity goal set for the project. The Odroid XU4 can then be easily replaced with a more powerful single board computer in case more computation capabilities are required.

A single board computer offers additional communication features that have also been exploited in this work. For instance, the camera can be controlled remotely by simply plugging a mobile network adapter to the board and through a tunnel take over the control from a remote personal computer (PC). This is specially useful when the camera is mounted onboard a UAV.

#### 2.1.3. Band Pass Filters

**Figure 3:**
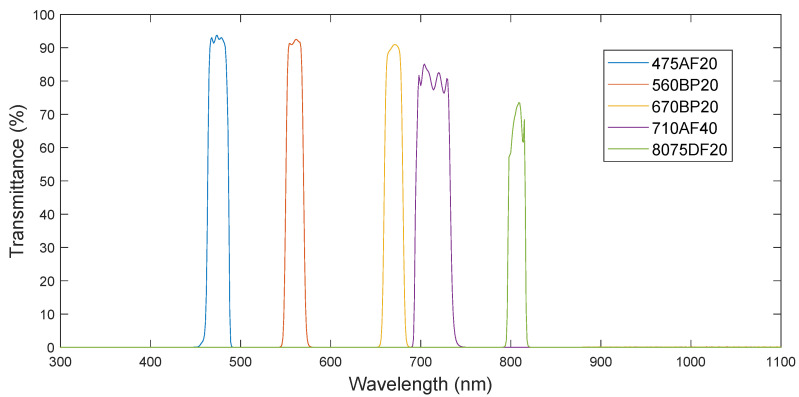
Filter transmittance curves.

The band pass filters are the crystal lenses placed in front of the optics that let radiation through at a specific wavelength and rejects the rest. As a matter of fact they feature a profile such as the one shown in Figure 3 with a narrow bandwidth and a transmittance, which represents the percentage of light the filters let through.

The camera has been designed so the installed filters can be easily modified and adapted to the needs of the application at hand. In this particular case, the ones that have been chosen are those providing a large set of vegetation indices. Filters have been acquired from the manufacturer Omega [51], which provided the details displayed in Table 3 for each individual filter.

#### 2.1.4. Filter Placeholders: Wheel Rotating System

In order to hold the filters and place the selected one at a time in front of the sensor, a rotating wheel has been designed. The wheel can contain up to six different band pass filters, although usually one of the placeholders is left empty so it is possible to capture a panchromatic image at a wavelength in the visible range of the spectrum. Additionally, having one empty placeholder would allow us to use more than one wheel, one on top of the other, multiplying the spectral resolution of the camera. This would require the addition of an extra motor but the controller would not have to be duplicated.

The wheel is set in motion thanks to a stepper motor controlled by the Arduino Nano in order to accurately position it at the desired angle. A limit or end switch sensor has been installed in order to get a feedback of the wheel position and increase accuracy even more. The mechanism of the sensor is simple, as it consists of a metal flap that closes an electrical circuit when the filter wheel reaches a certain position. During the camera initialisation process, the wheel starts automatically rotating until the limit switch sensor is detected so the exact position of the wheel is known from there onwards.

The Arduino Nano uses the general purpose digital outputs to control the stepper motor driver module that then transforms the signals into the necessary analogue values to drive the motor.

The advantage of having all processes centralised in the Odroid XU4 is that the image capturing and wheel positioning are perfectly synchronised. Before a capture takes place, the main process signals the Arduino Nano to move the filter wheel to the desired position and wait for the confirmation. Once the wheel is correctly placed, the main process triggers the capturing command.

The Arduino is directly powered through the USB connection from the Odroid XU4, which in turn is supplied from a converter that outputs a voltage of around 5 V and a maximum current of 3 A. This converter can be fed by a battery or any external supply that is within the voltage range from 8.4 V to 22.8 V. The motor is directly supplied from the output of the converter as well. The reason why it is not supplied from the Arduino is because it could eventually draw a high current value that exceeds the limitation of that board.

#### 2.1.5. Mechanical Assembly

**Figure 4:**
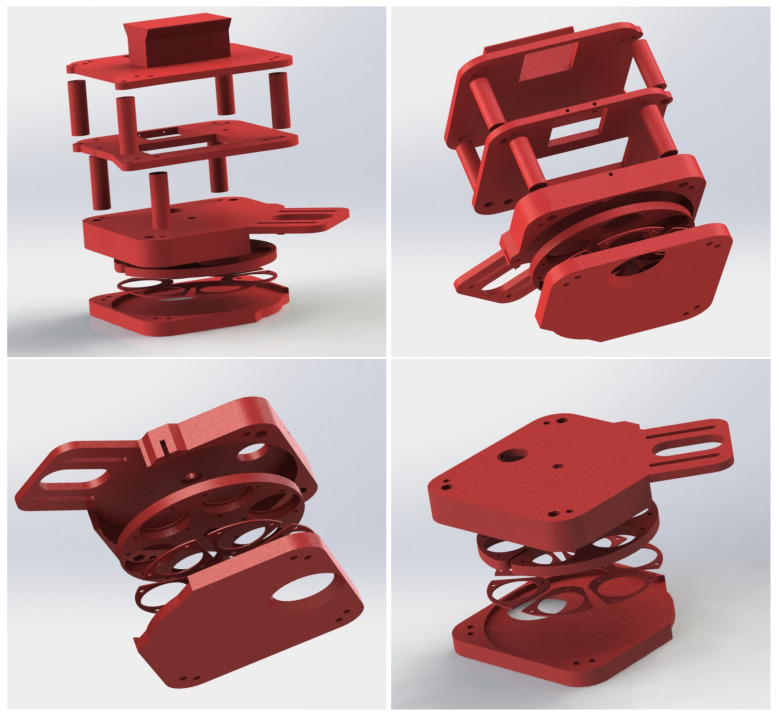
3D model of the camera structure.

The mechanical structure of the camera that holds all the components together has been designed with computer aided design (CAD) software and prototyped using a 3D Ultimaker (Geldermalsen, Netherlands) 3 Extended printer. Figure 4 shows the developed design from four different perspectives. As can be seen, the entire structure has been designed in three levels that have then been stacked together. The first level bears the rotating wheel and the necessary parts to make the motion mechanism work smoothly. The second level holds the IDS camera containing the sensor and the lens and the stepper motor. The third level houses the single board computer. Finally, on top of the third level, an element has been screwed to mount the camera into the carrying platform.

The proposed prototype is about 8.5 cm × 10 cm × 10 cm and weighs approximately 300 g. These dimensions satisfy the requirements for using the camera onboard a drone. The height of the modules can be adjusted to fit other components, such as an external battery, in case it can not use the drone power supply. The camera can be directly hooked to the drone structure or ideally be mounted in a mechanical stabiliser that dampens the vibration of the carrying platform. The high resolution of the sensor provides enough spatial information to perform image registration and correction algorithms for a later mosaic composition.

**Figure 5:**
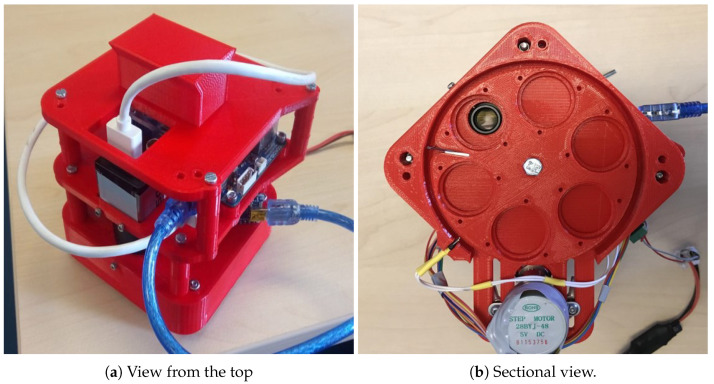
Views of the final camera assembly.

The final assembly, including all mentioned components, is displayed in Figure 5. In Figure 5a, a view from the top is displayed, where the IDS sensor located on the side with some cables and the element to hook the camera to a carrying platform, are visible. Figure 5b shows a sectional view of the camera in which the bottom lid has been removed, to show the wheel with the filter placeholders, the end switch, the optics of the sensor and the stepper motor.

### 2.2. Software Development

The previous section covered in detail the hardware elements involved in the proposed multispectral camera. The next step in the integration process should take care of coordinating all these elements together in order to capture a multispectral image made up of five individual captures. This coordination is carried out with a set of software implementations that are described in this section.

The developed software can be divided in the following applications:

- IDS camera control application running on the Odroid XU4, programmed in C++ and based on the IDS uEye SDK [52];
- Wheel rotation control application running on the Arduino Nano, programmed in Arduino programming language and supported by the Stepper and Serial libraries [53,54];
- Main application running on the Odroid XU4, programmed in C++ and supported by the Linux Serial library [55].

The first application consists of a set of basic functions to properly interact with the sensor, enhancing and adapting the functions already available in the sensor API to the multispectral camera needs. This means first receiving the camera parameter values from a master application, such as exposure time, brightness, gain levels or frame rate, and properly setting them to the sensor, taking into account its limitations. It must be highlighted that some of these values are user defined and others are obtained during the calibration phase, which will be further explained in Section 3, but they should all remain the same throughout the whole mission. Secondly, this involves receiving a trigger signal again from a master application and issuing that signal to the sensor in order to capture a single frame. Finally, the last application comprises storing that frame in the defined location in memory. The developed application was programmed with a public interface in C++, so the functions can be called from anywhere by just importing the header. This is especially relevant when using other sensors different than the IDS camera, because in that case the main application is fully decoupled from this camera application, which is in charge of calling the specific functions from the sensor application programming interface (API). As explained in Section 5, other sensors have been tested in order to assess different performances. For that purpose, the IDS uEye SDK, as well as the Video for Linux v.2 (V4L2), have been integrated.

The second application is in charge of the wheel rotation and positioning of the filters in front of the camera lens. It is programmed in the Arduino programming language and implements a simple interface to interact with the main application running in the single board computer. The Arduino software receives the commands from the main application via the serial port, in which it is specified which motor to move (the software is prepared for a case in which more than one motor is installed), and to which specific position. As already discussed in Section 2.1, in order to increase the accuracy in the positioning of the stepper motor, an end switch was added to the system. This switch is connected to the Arduino digital inputs and it is used to signal when the start or zero position has been reached.

The main process application is the one that coordinates and synchronises the sensor capturing with the filter wheel positioning. This software is responsible for initialising the camera and the filter wheel by bringing it to the start position, configuring the images’ output folder and controlling when to capture those images, in which format, and which filters to use. All configuration parameters are written in an extensible markup language (XML) file that is then loaded by this main application. In order to perform the capturing process, the main application can be either run in manual mode and then through the console the user issues the commands or it can be controlled automatically using the share memory space in Linux. The main process will read a certain space in the system share memory to know when to start and when to stop capturing images. This way, the capturing process can be automatised during, for instance, flight missions, when the camera is hooked up to a UAV.

**Figure 6:**
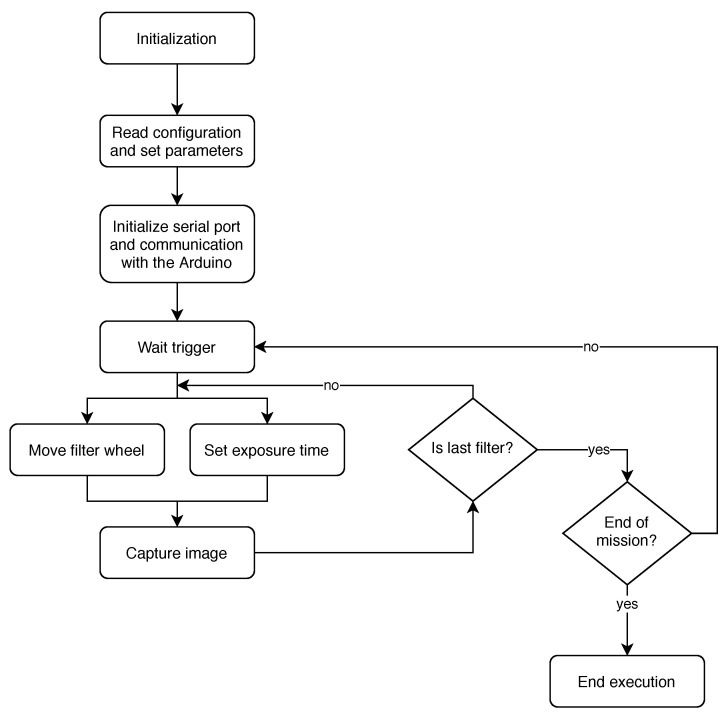
Main Application Diagram.

Figure 6 displays a software diagram of the main application. First, the camera is initialised, the image sensor is started and the wheel is automatically brought to the initial position using the end switch sensor. Once the wheel is in a known position, the main process proceeds to read the configuration file. Then, the calibration file is read, where the optimal exposure times for each individual filter are stored. Once everything is correctly configured, the camera enters an idle status in which it waits for the trigger signal to start capturing images. This can be triggered by the drone flying software or manually by the user that is connected to the camera remotely. Once the start has been issued, the camera proceeds to position the wheel to capture with the first filter, then it sets the exposure time of the sensor to the corresponding one and next it captures the image. Later it will move the filter wheel to the next filter to be used and repeats the process until single frames have been captured with all desired filters. After that, if the stop command is issued, the application will end, otherwise it will go back to the idle mode, waiting for another trigger.

## 3. Calibration Methodology

In Section 2.1.3, the transmittance of each individual filter is presented. As can be appreciated there, the shapes of the individual curves are quite different from each other both in width and in height. This means that the individual filters have a dissimilar response to light radiation. As a matter of fact, a filter with a higher bandwidth will irremediably let more energy pass through, as opposed to a filter with a narrower bandwidth, which blocks more radiation, obtaining darker images if the same exposure time was to be used. Therefore, in order to avoid capturing saturated images or images that do not use as much pixel dynamic range as possible, the exposure time of the camera has to be calculated for each individual filter.

**Figure 7:**
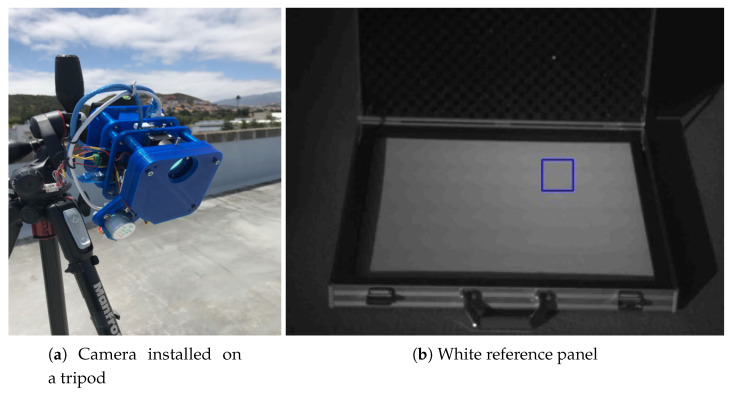
Calibration process.

This is what is called camera calibration and it is essential to have the multispectral camera perfectly set up to obtain images that can be correctly interpreted. The process is performed prior to the flight with the same illumination conditions that would be available during the mission. Otherwise, on sunny days we could end up having saturated pixels, or in low light days, a dark image—in both cases causing a loss of information. In Figure 7, the calibration process is depicted with two images. The image on the left, displayed in Figure 7a, shows how the camera is mounted on a tripod pointing downwards to capture an image of a certified Zenith Polymer white panel that reflects more than 99% of the incident radiation in all the VNIR range [56], displayed in Figure 7b.

A central area of the captured image, represented with a blue bounding box placed somewhere in the middle of the image, is automatically selected. The values of the pixels inside the selected sample area are compared with a target value, which in this particular case has been set to 95% of the captured image pixel depth, in order to ensure a short margin. Pixel values above that threshold are considered to be saturated. This criteria is fundamental in the calculation of the optimal exposure time.

**Figure 8:**
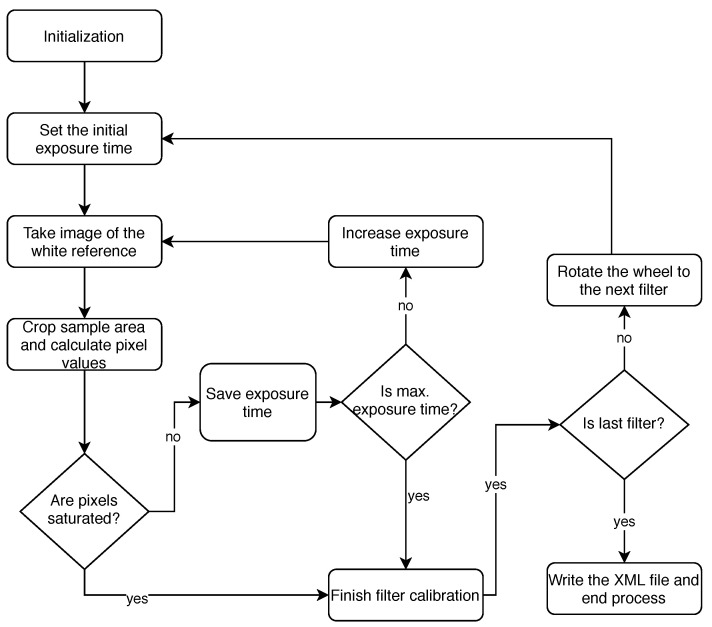
Calibration Diagram.

Summing up, each filter must have a different exposure time, which also depends on the environmental conditions of the particular date and time. Thus, given the particularities of the designed camera, a C ++ program has been developed to automate the entire calibration process and be able to perform it every time a mission is going to be executed. The methodology is simple and it is presented in Figure 8.

First, the program initialises the camera and checks that all the connections are in place. Then, the camera proceeds to start the calibration with the first filter. The procedure consists on performing a sweep through the exposure time range given in the specifications of the sensor. It starts by setting the exposure time to the minimum value, and capturing ten images of the white reference panel that are later averaged. Then, the resulting image is cropped, as explained earlier, and the values of the pixels inside the sample area are compared to the pre-selected target value. If more than 90% of the pixels values are below the threshold, the exposure time is increased and the capturing process performed again. In each iteration, the optimal exposure time is updated as long as the aforementioned criteria are fulfilled. Selecting the exposure time that provides the closest pixel value of the white reference without saturating the image ensures that any image captured afterwards with this filter and the selected exposure time will not saturate. Once the sample area pixels saturates, the process is terminated and optimal exposure time value stored.

This methodology is repeated with every filter and the appropriate exposure time for each one stored in an XML file to be used during the mission. In the case that a calibration process for a filter fails, the average value between the minimum and the maximum exposure time will be used with this filter to try to minimise the possible information loss at any weather conditions. Furthermore, knowing the reflectance response of the white reference panel, the raw values captured in the individual captures with each filter can be converted to reflectance values, applying the formula defined in Equation (1). In the formula, *sensed_bitarray* are the 2D raw data of the scene captured by the sensor, *dark_reference* represents the noise of the electronics in the sensor when data are captured with a shuttered lens and *white_reference* is the already mentioned reflectance response of the white reference panel. Equation (1) is applied pixel-wise as all involved data are two-dimensional, and for each captured band in the multispectral image. The resulting *reflectance* is a 3D image with values spanning from 0, which represents very low reflectance of the captured scene, to 100%, which represents a very high reflectance of the scene.

(1) $$reflectance\left(\%\right)=\frac{sensed\_bitarray-dark\_refence}{white\_reference-dark\_reference}\times 100$$

As mentioned before, the ideal exposure times are individual to each filter. Therefore, it is necessary to perform the calibration process for every new filter to be used, as well as when the light conditions at which the captures take place vary.

## 4. Characterisation Methodology

The characterisation process aims to quantify the spectral performance of a camera, in terms of accuracy in the acquired radiation values per wavelength. However, when dealing with different sensors, this task is not straight forward, since each sample is measured according to a local reference system, which very much depends on the characteristics and design of the specific sensor. For this purpose, the way the amount of energy reflected or absorbed by the different elements in the scene is interpreted, is by comparing them with the maximum and minimum amount of energy the image sensor is capable of capturing. In order to obtain those values, the same reference panel displayed in Figure 7b has been used to sense the maximum amount of energy the camera is able to capture for each individual filter. The minimum amount of energy is simply obtained by shutting the lens.

Furthermore, in order to characterise the multispectral camera, an additional certified polymer is used with a spectral response that lies in between the minimum and maximum values mentioned before. This polymer supports the process of measuring the accuracy of the camera when capturing at different wavelengths, as the expected value is known in advance. The error is calculated as the difference between the obtained spectral value, measured by the camera, and the certified one for each specific wavelength.

The measured value is obtained by performing a capture with the camera in a situation with constant and controlled light conditions. This is the case of our laboratory, where a seal cage made of anti-reflective material is available, illuminated with Headwall Photonics light, consisting of a halogen bulb with a relatively homogeneous spectral emitting signal. The camera is mounted on a rail at the top of the cage, pointing downwards where the polymer is displayed. Once the capture has been performed and stored in memory, the pixels corresponding to the polymer are manually selected with a post-processing tool and averaged. The reflectance value of the calculated average value is obtained by applying the formula shown in Equation (1).

The theoretical or expected value is calculate, taking into account the spectral response of the components involved in the acquisition. That is, the response of the material itself, given by the manufacturer, and the transmittance curve of the filter. The latter is always lower than 100% at every wavelength, as displayed in Figure 3. Thus, the reflectance value of the certified material has to be scaled to correctly compare this value with the measured one. Furthermore, although the measured value is assigned to a specific wavelength, the centre value of the transmittance curve of the filter and the band pass shape response of the curve features a bandwidth, which needs to be taken into account as well. This means that not all the energy captured by the sensor corresponds to a unique wavelength in which the filters are centred, but to the whole range spanned by the transmittance shape.

The formula applied to obtain the theoretical value is shown in Equation (2), where $P_{i}$, is the reflectance value of the polymer at each wavelength, provided by the manufacturer of the material, $F_{i}$, the transmittance value of the filter at each wavelength and $WR_{i}$, the theoretical maximum value that can be sensed by the device at each wavelength, obtained from the certified spectral signature of the white reference material that reflects 99% of the incident light.

(2) $$T.V.=\frac{\sum_{i}F_{i}P_{i}}{\sum_{i}F_{i}WR_{i}}$$

**Figure 9:**
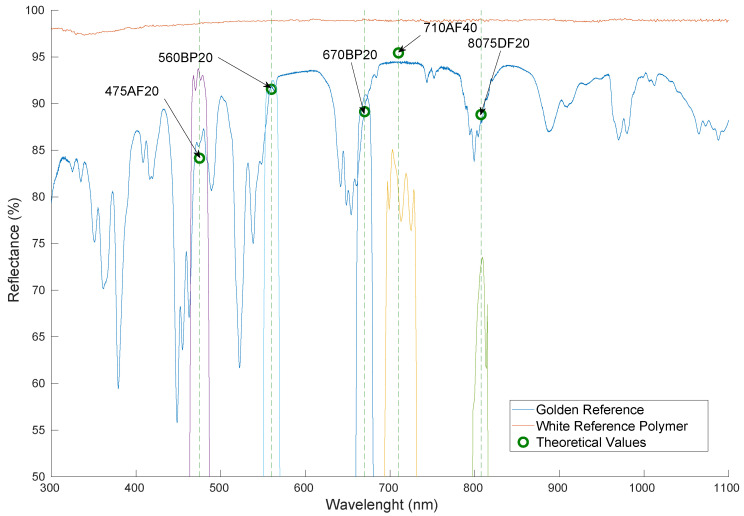
White and golden references with the theoretical values calculated for each individual band pass filter.

The division by the maximum reflectance is performed in order to be able to compare the value with the measured one, which also considered the maximum and minimum reflectances to be measured by the camera. In Figure 9, all mentioned spectral data required for the characterisation process are plotted. In red and blue, the spectral response of the white and golden references polymers curves are displayed. The theoretical values calculated using Equation (2) are represented as the green small circles for each band pass filter. As can be seen, all values lie very close to the golden reference spectral signature at the given wavelength. Finally, the transmittance shape of each individual filter is also represented in the figure.

Once the theoretical value has been calculated, it is compared with the captured value by the camera at different exposure times, in order to find the time range in which the sensor behaves more steadily. The error between both values is computed in the entire exposure time range for which the saturated images with high exposure times as well as the dark images with very low exposure times are discarded so they do not distort the obtained error. Ten images are captured with each exposure time, and then averaged to correctly characterise the existing error between both values. Table 4 details the differences between the theoretical values calculated following the methodology described in this section, and the measured values obtained with the developed camera.

The presented characterisation methodology let us compare the performance of the camera when using different sensors, as shown in Section 5. We use this characterisation methodology to quantify the performance differences between using the theoretically superior NIR sensor with other CMOS sensors.

## 5. Results

The results of the carried out work are split into two subsections. First, the characterisation of the camera is carried out using the NIR sensor that was selected to be installed in the multispectral camera. Its performance is compared against a much lower cost sensor with an red-green-blue (RGB) pattern. These tests are done in the laboratory, where the light conditions available from a source with a well known emitting signal, let us capture multispectral images in a controlled environment, hence, obtaining reflectance values at the different wavelengths that can be correctly compared with each other and with the certified material.

Secondly, the camera is used to uncover vegetation index results, both from images captured in the laboratory as well as images obtained from a flight mission performed with the camera hooked up to a drone.

### 5.1. Sensor Performance in the Laboratory

This section summarises the results obtained during the characterisation process. The methodology has been applied to the camera already mentioned in Section 2.1.1, CMV2000-3E12M CMOSIS sensor. Additionally, the process has been replicated with another sensor available at our laboratory, the Sony IMX291 (Minato city, Tokio) [57] sensor with an RGB filter pattern, integrated into the camera SUSB1080P01 [58] from the ELP series (Ailipu Technology Co., Shenzhen, China) [59]. The reason for doing this comparison is to show the performance difference between an NIR sensor and a one that features an RGB filter pattern on top, which has a considerably lower price. Furthermore, this type of sensor is indeed often found in the cameras available in the market. As already mentioned, the main cost driver of the multispectral camera is the sensor; hence, if in the future it is desired to decrease its overall cost, one way would be by replacing the sensor. Nonetheless, as it will be shown in this section, this comes with a considerable performance loss.

**Figure 10:**
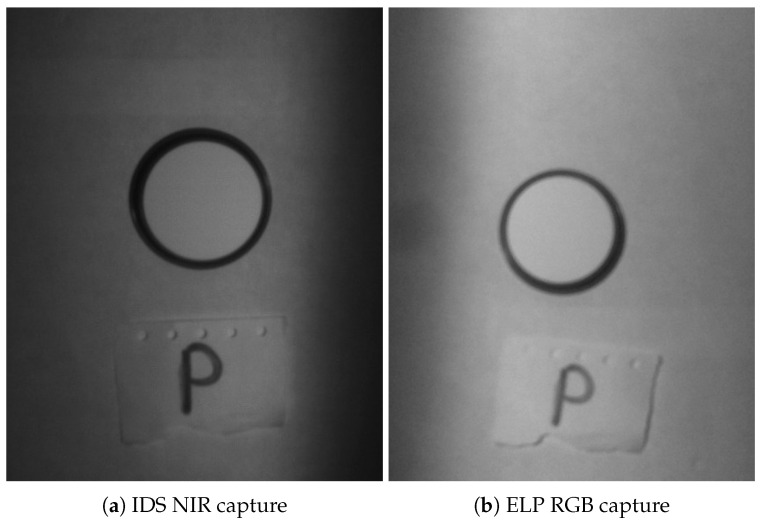
Golden reference polymer captures.

In order to make this comparison, different aspects have been taken into account. First, the characterisation process described in Section 4 is accomplished for both sensors, having already calculated the theoretical values of the golden reference polymer. The captured values for each sensor and filter have been achieved by performing an acquisition of the material at the exposure time obtained during the calibration process, giving the optimal exposure time for each band pass filter. Figure 10 shows an individual capture of the golden reference polymer taken with the NIR sensor, represented in Figure 10a, and with the RGB sensor, represented in Figure 10b. A rectangular area inside the polymer is manually selected with the aid of a graphical user interface tool and then, the pixels within that area are averaged. For the particular case of the RGB sensor, the image is converted to luminance and chroma components (YCbCr) to just retain the brightness and proceed as if it was a panchromatic image.

Finally the average value is normalised using the white reference, captured at the same exposure time and for each filter and sensor as well. Table 4 displays the theoretical and calculated values (C.V.) and offers a comparison against each other in percentage values.

As can be seen, the IDS sensor provides an overall better performance, only giving a higher error rate for the first filter centred at 475 nm (*475AF20*). At this wavelength, the source of light at the laboratory has less emitting power and this could have influenced the obtained result. Moreover, a visual inspection of the acquisitions made by each sensor displayed in Figure 10 lets us see a quality difference between the images captured, with the one acquired with the RGB sensor showing less sharpness.

**Figure 11:**
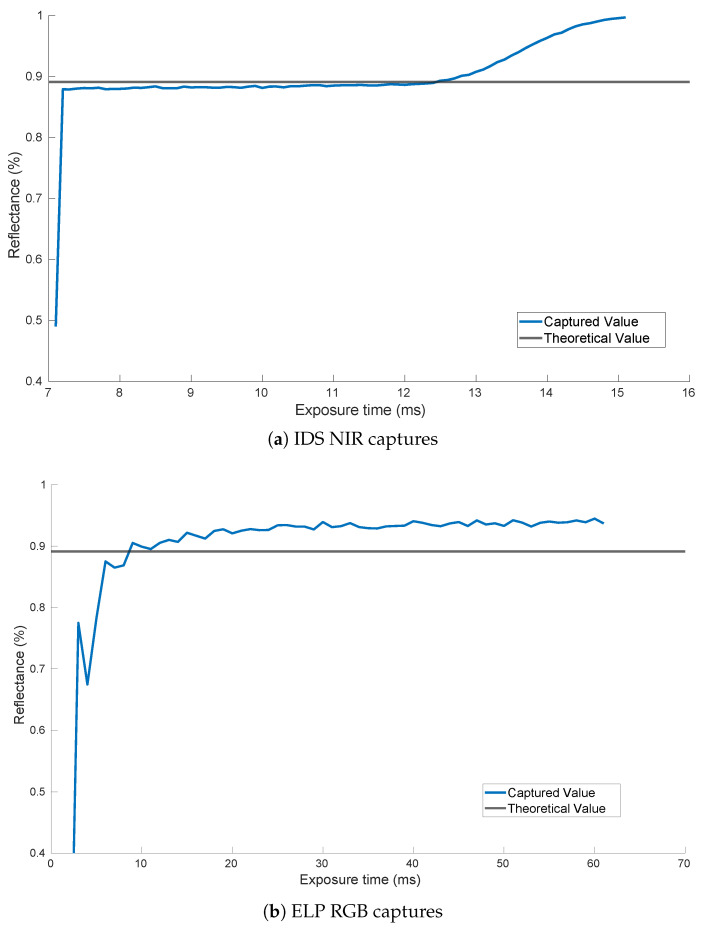
Results of the exposure time swept with both sensors.

The second feature that has been inspected in the comparison is the stability of the results for a wider range of exposure times and not just the optimal one, obtained in the calibration process. For that purpose, an exposure time sweep was carried out so that the captures started a few milliseconds before the optimal exposure time and ended a few milliseconds after. Figure 11 shows the result for filter *670BP20* with both sensors. It is important to notice that the range of both figures is not the same as the IDS UI-3360CP camera, integrating the CMV2000-3E12M sensor, which allows for a precision of microseconds when setting the exposure time, while the ELP SUSB1080P01 camera integrating the Sony IMX291 sensor allows only a precision of milliseconds. Therefore, in order to show the same number of exposure time steps, the time range is different in both cases.

The curves displayed in Figure 11 show a much smoother result for the sensor integrated in the IDS camera. For a range that spans from 7.2 ms to 13 ms of exposure time, the captured value lies very close to the desired value. On the other hand, the sweep performed with the Sony IMX291 sensor shows a significant offset between the captured values and the theoretical one. The reason for performing this analysis lies in the fact that, in the calibration process, it is not always possible to achieve the optimal exposure time value; therefore, having a range in which the sensor behaves very similar indeed constitutes an advantage.

Overall, the NIR sensor shows a better performance than the RGB sensor. Additionally, it offers better features, such as higher frame rate, higher resolution and higher signal to noise ratio (SNR). On top of that, the IDS camera provides an SDK that enables a fine control of all acquisition parameters and lets the user obtain the captured images in different formats from raw captured values, to compressed formats, such as joint photographic experts group (JPEG). For all the mentioned reasons, it was decided to use the NIR sensor in the multispectral camera presented in this work. The results presented from here on in this manuscript were acquired using that sensor.

At this point, it is worth making a comparison between the proposed camera and one of the golden references available on the market, the RedEdge-MX (Micasense, Seattle, United States of America) [18], widely used by the scientific community in numerous experiments. As has already been discussed, one of the main advantages of the proposed solution is its flexibility and modularity, which makes it adaptable for different projects and scenarios, whereas the RedEdge-MX is a closed solution with a predefined set of filters. Another interesting aspect of the developed camera is the possibility of having the captured data available right away, an instant after its acquisition, a feature of key importance in real time applications, while the RedEdge-MX comes with a memory that is retrieved after the mission to access the captured data. Finally, the overall cost of the proposed solution, below EUR 2000, is considerably lower than the RedEdge-MX, around EUR 5000.

### 5.2. NDVI Results

One of the immediate results that can be obtained from the images captured with the multispectral camera is the generation of a set of vegetation indices (VIs) which provide characteristic information about the status of the crop [60], such as health condition, hydrogen concentration, and water stress, among others. This is performed by combining or transforming two or more spectral bands designed to enhance the contribution of vegetation properties, allowing for reliable spatial and temporal inter-comparisons of terrestrial photosynthetic activity and canopy structural variations.

In particular, the well known normalized difference vegetation index (NDVI) [61] provides an orientation of the health condition of the plant by measuring the difference between near infrared (which vegetation strongly reflects) and red light (which vegetation absorbs). The formula of the NDVI is displayed in Equation (3).

(3) $$NDVI=\frac{NIR-Red}{NIR+Red}$$

Theoretically, this spans the result in the range {−1,1}. However, in most elements the NIR reflection is higher than the red and therefore, the practical results tend to be positive or, when negative, very close to 0. In this Section, all results have been normalised to be spanned in the range {0,1}.

The light filters installed in the camera and presented in Table 3 have provided a designation equivalent to the variables used in the formula. In this particular case, frames captured with filter *670BP20* and filter *8075DF20*, which correspond to the red (≈670 nm) and the NIR (≈800 nm), respectively, are being used.

The developed camera was used to calculate the NDVI index of the scenes captured in two different experiments. The first one took place in the laboratory with the camera fixed to a tripod. The staged scene is a composition of different elements, plants and other non-plant objects with a greenish aspect, in order to check the response differences of the living and inert objects in the NDVI results. The scene was illuminated with an halogen source of light.

**Figure 12:**
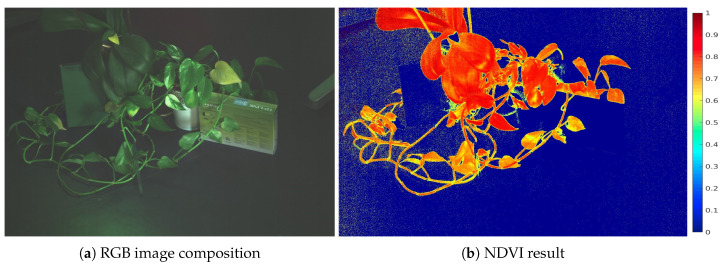
Images captured in the laboratory with the multispectral camera.

In Figure 12, the results of the experiment are displayed. In Figure 12a, the Blue, Red and Green captured bands have been used to display a false RGB image of the scene. In Figure 12b, the NDVI result is displayed. As appreciated all inert objects have provided a value close to 0 and are therefore represented in blue colour. On the other hand, the plant is highlighted in red, corresponding to values close to 1, and hence healthy living matter.

**Figure 13:**
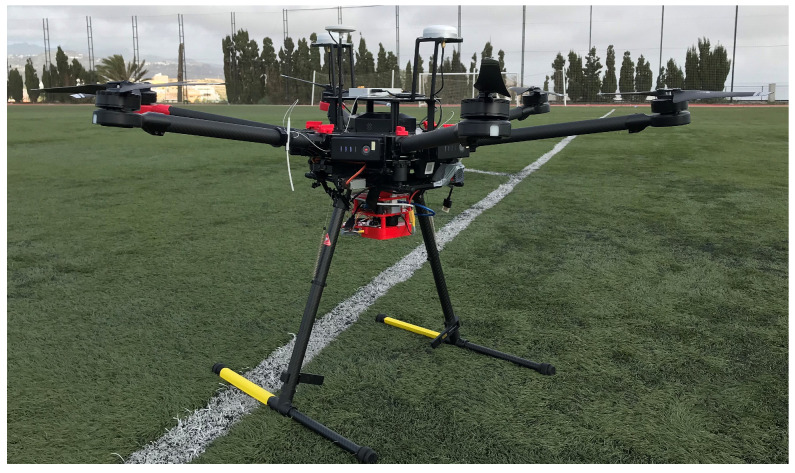
The multispectral camera installed in the Dji Matrice 600.

The second experiment consists in capturing images with the camera from the air, as the ultimate purpose of its design is to be installed in aerial platforms. For that reason, a hook with a dovetail shape was added on top of the structure to easily mount the camera on a drone and have it always pointing downwards. The selected platform to carry the device in this case was the Dji Matrice 600 [62], that is available at our facilities and has already been used to perform missions carrying other types of sensors. The advantage of using that platform is that the integration is relatively straight forward, as the software that plans the mission also controls the flight and synchronises it with other processes, which was already developed in a previous work [63]. Figure 13 presents the UAV platform with the camera hooked underneath it, ready to start a flight mission.

**Figure 14:**
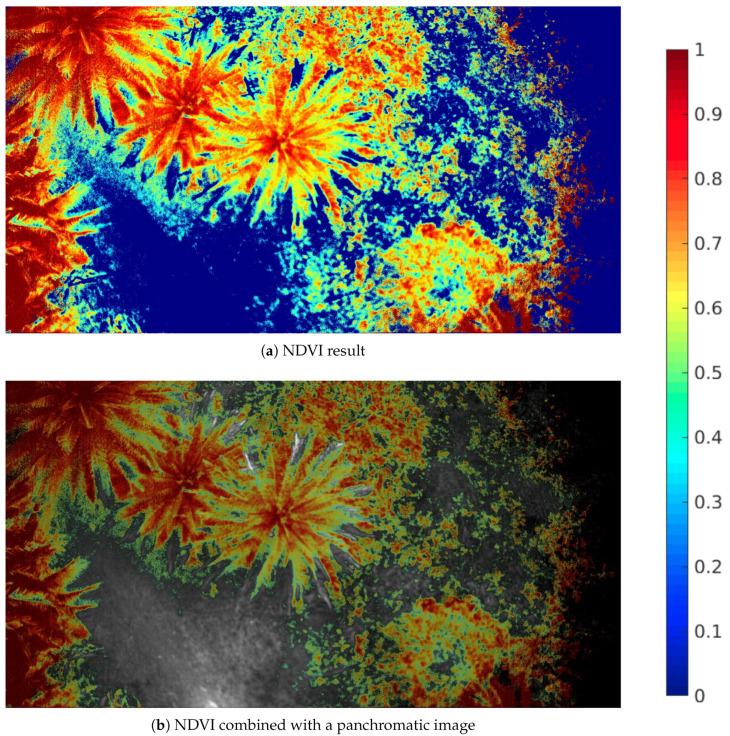
Results of one of the flights performed at the University campus, over a palm tree plantation.

The area of choice for performing the flight tests was a football field and its surroundings located at the University of Las Palmas de Gran Canaria campus. GPS coordinates of the location are 28°04’17.9” N 15°27’25.9” W. For this test, the data were captured following a fixed-point hovering strategy. Nonetheless, the system is prepared to carry out flight missions as well. In this case, the user selects an area to be scouted and the application calculates the intermediate waypoints based on inputs such as image overlapping, flight height, etc. In this way, multiple multispectral images are then captured between waypoints. Flight speed is limited by the maximum frame rate of the camera, which depends on the user inputs as well. In Figure 14, the results obtained during one of the performed missions over a palm tree area are presented.

Figure 14a shows the NDVI colour map generated from the captured information by the developed camera. Once again, vegetation tends to have a red colour, while background is represented in blue. Figure 14b combines the NDVI colour map result with the panchromatic image of the same area. The filter wheel contains six placeholders and just five of them are filled with filters. The extra hole is left empty in order to capture an image with no filter of the area. This image is being used here as a background and on top of that, the NDVI result is being displayed after removing the uninteresting part (blue colour pixels in Figure 14c). This way, the panchromatic image is enhanced with the vegetation index result.

## 6. Conclusions

In this work, a low cost and modular multispectral camera based on a single panchromatic sensor and a moving wheel of interchangeable band pass optic filters is presented. Design aspects related to mechanics, optics, software, hardware and 3D modelling are detailed showing the importance and synergies of this multidisciplinary research. Moreover, a calibration and characterization methodology are developed and presented in order to ensure the acquisition process and the feasibility of the obtained images. The camera was mounted onboard a Dji Matrice 600 UAV in order to validate and quantify its performance for the particular case of precision agriculture, for which coloured NDVI maps were generated for a palm tree plantation area. The system is presently in a pre-commercial phase as further tests are being carried out in order to test its feasibility to support farmers willing to monitor their crops with a reduced amount of time and effort. Another interesting area that is currently being explored is environmental research by simply changing the set of optic filters being used, an easy task due to the modularity advantage of the solution.

## Figures and Tables

**Table 1:** Camera and sensor specifications.

Camera	Model	IDS UI-3360CP
	Max. Frame Rate [fps]	152
	Exposure time (min–max) [ms]	0.025–500
	Optics	3.5mm HR
	Interface	USB 3.0
	Weight [gr]	52
Sensor	Model	CMV2000-3E12M
	Type	CMOS NIR
	Resolution	2048 × 1088 Pixels
	Optical Format	2/3”
	Pixel Size [μm]	5.5
	Max. Frame Rate [fps]	340
	Output Interface	LVDS 16x 480Mbps
	Shutter Type	Global

**Table 2:** Odroid XU4 sigle board computer specifications.

Chip	Samsung Exynos 5422
CPU	2.1GHz Quad-Core (Cortex-A15) and 1.4GHz Quad-Core (Cortex-A7)
GPU	Mali-T628 MP6 (OpenGL ES 3.1/2.0/1.1 and OpenCL 1.2 Full profile)
DRAM	2Gbyte LPDDR3 RAM PoP stacked
Storage	eMMC5.0 HS400 Flash Storage
Interfaces	2 × USB 3.0 Host, 1 × USB 2.0 Host
	Gigabit Ethernet
	HDMI 1.4a for display
Size	83 × 58 × 20 mm approx. (excluding cooler)
Power Supply	5 V/4 A input

**Table 3:** Omegafilters bandpass filters implemented in the camera.

Filter ID	Designation	Center Wavelength (nm)	Bandwidth (nm)	Transmittance (%)
475AF20	Blue	475	20	≥92
560BP20	Green	560	20	≥85
670BP20	Red	670	10	≥85
710AF40	Rededge	710	10	≥85
8075DF20	NIR	807	20	≥60

**Table 4:** Comparison between theoretical and obtained values for each sensor and filter.

Filter ID	Theoretical Value (%)	IDS Sensor		ELP Sensor	
		C.V. (%)	Error (%)	C.V. (%)	Error (%)
475AF20	84.15	88.05	4.64	85.44	0.015
560BP20	91.53	91.52	0.02	91.16	0.41
670BP20	89.13	89.15	0.015	89.7	0.63
710AF40	95.43	95.38	0.16	97.41	2.07
8075DF20	88.82	88.83	0.018	94.02	5.84
